# *Escherichia coli* recombinant sperm immobilizing factor RecX as a potential vaginal contraceptive

**DOI:** 10.1186/s12958-018-0407-1

**Published:** 2018-09-13

**Authors:** Monika Answal, Vijay Prabha

**Affiliations:** Department of Microbiology, Panjab University, Chandigarh, 160014 India

**Keywords:** Vaginal contraceptives, Reproduction, Sperm, Cloning, Sperm immobilizing factor, Contraceptive agents, Spermicidal, Fertility control

## Abstract

**Background:**

To control the overpopulation and unintended pregnancies, vaginal contraceptives have gained recent surge of interest because of its topical application with possible avoidance of systemic effects. However non-specific cytotoxicity associated with detergent-based synthetic vaginal contraceptive agents limits their use and generates considerable interest in the development of vaginal contraceptives of biological origin for controlling reproduction and ultimately growing population. In this study, we have cloned, over-expressed an *Escherichia coli* gene encoding a sperm immobilizing factor (SIF) that inhibits sperm motility for the development of vaginal contraceptive from a biological source i.e. *E. coli*. The contraceptive efficacy of the *Escherichia coli* recombinant sperm immobilizing factor (r-SIF) was also determined.

**Methods:**

Genomic DNA library of an *E. coli* strain isolated from semen sample of an infertile male was constructed for the identification and cloning of *E. coli* SIF coding gene. This gene was sub-cloned in pBADmycHisB for over-expression and the r-SIF was purified using Ni-NTA affinity chromatography. Effect of r-SIF on mouse sperm motility, viability and on morphology was evaluated. Binding of r-SIF to mouse sperm was demonstrated by fluorescent labeling. Contraceptive efficacy of r-SIF was checked in murine model.

**Results:**

Genomic library resulted in five hundred transformants; five clones were found positive for sperm immobilizing activity. The protein product of the insert DNA sequence in one of the transformants showed maximum sperm immobilizing activity. Sequence analysis of ORFs in the insert revealed homology to *recX* on both nucleotide and protein level. 40 μg of the purified r-SIF showed immediate spermicidal activity in vitro for mouse sperm. Scanning electron micrograph of the r-SIF treated sperm showed intense morphological damage to sperm. FITC labeled r-SIF showed highest fluorescence at the head region of the sperm. 5 μg of purified r-SIF exhibited a complete contraceptive effect in mouse model.

**Conclusion:**

r-SIF could be seen as potential target to be developed as potent and safe vaginal contraceptive in future.

## Background

During the twentieth century alone, the population in the world has grown from 1.65 billion to 6 billion and is projected to touch 9.4 billion by 2050. Population explosion and a higher rate of unintended pregnancies are affecting the resources worldwide [1]. Contraception is an essential component of preventive measures that allows women to have planned pregnancies that ultimately lead optimal health, social and economic outcomes for women, families and society. However available contraceptive methods still have associated side effects, failure rate or irreversibility [2]. Oral contraceptive pills have highest contraceptive efficacy and reversibility, but these sex-steroid based pills are not without risks [3]. Intrauterine devices carry the possibility of expulsion and upper genital tract infections [4]. Contraceptive efficacy of condoms is compromised by their improper and inconsistent use [5]. Their use is coitus-related and depends on the co-operation of a male partner. Men being reluctant to use condoms and women being unable to negotiate with a partner; limits the use of this simple option of fertility control. Cultural biases and improper use or breakage during intercourse are some other compromising issues related to the efficacy of condoms [6]. Other approaches such as emergency contraception are not popular due to high cost and requirement of clinical supervision to certain extent [7]. Natural family planning is not sure and safe as many couples find them difficult to calculate safe period for the copulation, especially in women with irregular menstrual cycles. Reliance on surgical sterilization increases with age and can be used by women who are certain about their desired childbearing. The whole playscript reflects that only 5% of unwanted births are due to contraceptive failure and remaining 95% above burden is from women who do not use any contraception, experience gaps in the use of contraception, or use contraception incorrectly or inconsistently [8]. To turn this enormity corner, scientific predilections should be inclined on new targets that might be exploited for the development of safe, effective and inexpensive contraceptive agents.

Vaginal contraceptives being topical agents avoid systemic consequences and can address many of the issues and fit in women agreement hence may give them the power to safeguard themselves. However, available vaginal contraceptive formulations such as nonoxynol-9 (N-9), sodium dodecyl sulfate (SDS) and benzalkonium chloride exhibit detergent-type cytotoxic effect on cervicovaginal epithelium and normal vaginal flora. Their repeated use renders the user to discomfort, vaginal irritation and more susceptible to sexually transmitted diseases, including HIV [9, 10]. These limitations encourage researchers to develop vaginal contraceptives from biological sources, e.g., Immotilin [11], Magainin [12] and Nisin [13] are being examined as spermicidal agents. Microorganisms are also known to block sperm motility either by agglutination or by secreting extracellular substances. Reports are also available for microbial spermicidal factors [14–17]. Likewise, we observed, our laboratory *Escherichia coli* strain was producing a sperm motility inhibiting factor i.e. sperm immobilizing factor (SIF). Potential of SIF to inhibit sperm motility in vitro strengthened its utilization for the development of new vaginal contraceptive of biological origin. However, further progress with SIF in its crude form was not possible. Therefore, to obtain SIF in pure form it was necessary to characterize it at molecular level. In the present study the gene encoding for SIF was cloned, over expressed and pure recombinant factor was obtained. The r-SIF was evaluated both in vitro and in vivo for its spermicidal activity and contraceptive efficacy respectively.

## Methods

### Bacterial strains, plasmids, and enzymes

Sperm immobilizing factor producing *Escherichia coli* was previously isolated in our laboratory from semen sample of an infertile male, from the Department of Urology, PGIMER, Chandigarh [18]. *E. coli* DH5α and *E. coli* BL21 cells used as cloning host and expression host respectively, were cultured in Luria-Bertani (LB) broth (Himedia Labs, India) containing ampicillin (100 μg/ml). Plasmid pSMART-LCAmp (Lucigen, USA) was used for the preparation of genomic library and pBADmycHisB (Invitrogen, India) used for expression of SIF. Restriction enzymes and T4 ligase were purchased from Thermo Scientific, India. Ni-NTA resin was purchased from Qiagen (India). The primers used were synthesized from Eurofins genomics, India (Table 1).

**Table 1:** Primers used in the study

Primer	5′-3′ sequence	Restriction site
recX F	5′-GTTGTAAGGATATGCCATGGCAGAATC-3′	NcoI
recX R	5′-GTATGCGGTCGACCGGCAAAATTTC-3′	SalI
SL1	5′-CAGTCCAGTTACGCTGGAGTC-3′	–
SR2	5′-GGTCAGGTATGATTTAAATGGTCAGT-3′	–

### Animals

Sexually mature, 5–6 weeks old male (26 ± 2 g) and 4–5 weeks old female (23 ± 2 g) Balb/c mice (Mus musculus) were used in the present study. The animals were housed in polypropylene cages and kept in the animal house of the Department of Microbiology, Panjab University Chandigarh. The animals were maintained in laboratory conditions (12:12, dark: light cycle), fed with standard pellet diet (Hindustan Lever products, India) and water ad libitum. All the experimental protocols were approved by Institutional Animal Ethics Committee of the Panjab University, Chandigarh, India, vide letter number PU/IAEC/S/15/71 dated 15.09.2015 and were performed in accordance with the guidelines of the Committee for the Purpose of Control and Supervision of Experiments on Animals, India.

### Construction of genomic library

Genomic DNA of *Escherichia coli* producing sperm immobilizing factor (Qiagen DNA extraction kit) was partially digested with HaeIII and the DNA fragments were separated by gel electrophoresis to obtain 2–6 kb DNA fragments which were purified from agarose gel (Qiagen gel purification kit). Genomic DNA fragments were cloned at the blunt ends of pSMART-LCAmp plasmid and transformed into *E. coli* DH5α cells using heat shock technique [19]. The transformants were obtained on LB agar containing 100 μg/ml of ampicillin and transferred to fresh LB-ampicillin plates.

### Screening of genomic library

Transformants were screened for sperm immobilizing activity. In brief, each of the transformants was grown separately in 50 ml LB broth supplemented with ampicillin and incubated at 37 °C/200 rpm for 14–16 h. Cell pellet obtained after centrifugation at 10,000 rpm for 10 min was re-suspended in 10 ml of phosphate buffer saline (PBS) pH 7.4 and sonicated (30% amplitude, 10 s on, 20 s off; total time 2 min). Cell debris was removed after centrifugation at 10,000 rpm for one hr. The motile population of sperm was obtained from vas deferens of mouse excised after the autopsy. Sperm were collected in physiological saline and motile sperm count was adjusted to a concentration of 40 × 10^6^/ ml. The cell lysate was mixed with mouse sperm in equal proportions and incubated for different time intervals, i.e., 5 min, 15 min, 30 min and 1 h and analyzed under a light microscope at 400X (Olympus India Pvt. Ltd) for sperm immobilizing activity. Cell lysate obtained from the culture of *E. coli* DH5α containing pSMART-LCAmp was also checked for sperm immobilizing activity. Plasmid from transformants that displayed maximum sperm immobilizing activity was isolated (QIAprep Spin Miniprep Kit) and DNA sequencing was performed for the determination of the insert sequence (Chromous Biotech India Ltd.).

### Sequencing of insert DNA fragment and bioinformatics analyses

Insert DNA fragment was analyzed for the presence of ORFs in all reading frames using ‘ORF Finder’ program (https://www.ncbi.nlm.nih.gov/orffinder/). Database homology searches of nucleotide and deduced amino acid sequences obtained were carried out using BlastX and BlastP (https://blast.ncbi.nlm.nih.gov/Blast.cgi) on the NCBI website. The primers to amplify the target gene were designed using ‘Gene runner’ software.

### Construction of an over-expression system

To over-express SIF encoding gene, the identified ORF region was amplified by PCR using primers RecX F1 (5′-GTTGTAAGGATATG**CCATGG**CAGAATC-3′) and RecX R1 (5′-GTATGCG**GTCGAC**CGGCAAAATTTC-3′) containing NcoI and SalI sites, respectively. For amplification, the PCR conditions were as follows: a hot start at 95 °C for 5 min, 30 cycles of 95 °C for 1 min, 56 °C for 1 min and 72 °C for 45 s, followed by one cycle of 72 °C for 10 min. The amplified PCR product was purified and digested by the restriction enzymes mentioned above and cloned into pBADmycHisB between NcoI and SalI sites. Cloning at SalI site ensured the presence of 6X histidine residues at C-terminal of the expressed protein. The cloned gene was transformed and expressed in *E. coli* BL21.

### Production and purification of recombinant *E. coli* sperm immobilizing factor (r-SIF)

*E. coli* BL21 transformant cells were cultured in 500 ml LB broth at 37 °C/200 rpm till absorbance at 600 nm (OD_600_) reached 0.5–0.6. The production of sperm immobilizing factor was induced by addition of 0.4% of L-arabinose (Sigma Aldrich, India) and subsequent growth for 4 h. Cells were harvested by centrifugation (7000 rpm, 20 min), re-suspended in lysis buffer [50 mM phosphate buffer, 0.3 M NaCl, 1 mM PMSF, 2 mM β-mercaptoethanol (pH 7.4)] and sonicated. Soluble proteins were separated from cell debris by centrifugation at 10,000 rpm for 1 h. Clarified cell supernatant was applied to 2 ml of Ni-NTA affinity column (Qiagen), preequilibrated with 15 ml of equilibration buffer (50 mM phosphate buffer, 0.3 M NaCl), followed by the column washing with 25 ml of Buffer A [50 mM phosphate buffer, 0.3 M NaCl, 10 mM imidazole (pH 7.4)] to remove loosely bound proteins. Recombinant protein containing histidine-tag was eluted from the column with Buffer A containing different concentration of imidazole (10–100 mM). Fractions containing tagged protein identified by SDS-PAGE were pooled and dialyzed overnight against sodium phosphate buffer (pH 7.4) at 4 °C and subsequently concentrated using Amicon concentrator (< 10 kDa) (Millipore). The protein concentration was measured by BCA protein estimation assay kit (Thermo Scientific, India) according to manufacturer’s recommendations. Molecular weight and purity of r-SIF were determined using 15% SDS-polyacrylamide gel with MiniProtean III system (Bio-Rad, India). The gel was stained with Coomassie Brilliant Blue R-250 and molecular weight was estimated with reference to the broad range molecular weight protein marker (Thermo Scientific, India).

### In vitro effect of r-SIF on mouse sperm parameters

- Sperm motility: Mouse sperm were mixed with different concentration of r-SIF (10–100 μg), after 20 s a drop of the mixture was observed on a microscopic slide under a light microscope at 400X. A control containing PBS mixed with mouse sperm was set up simultaneously. Complete immobilization of motile sperm within 20 s was recorded as a positive result and the corresponding highest dilution was taken as the minimum effective concentration (MEC).
- Viability: Mouse sperm viability was checked with Eosin staining method. Mouse sperm were mixed with PBS and MEC of r-SIF which served as negative and positive controls, respectively. After 20 s two drops of 1% Eosin were added to each and mixed well. A smear prepared from the mixture on a clean glass slide was observed under light microscopy at 400X to ascertain pink stained headed dead sperm from unstained headed live sperm.
- Sperm morphology: Scanning electron microscopy (SEM) was done to study the effect of r-SIF on the morphology of mouse sperm using Jeol scanning microscope (JSM-6100, SM Jeol 20 kV, Japan) by standard method [20] with slight modifications. Briefly, mouse sperm suspension was centrifuged at 500 rpm for 10 min, mixed with PBS as control and MEC of purified r-SIF as test; both incubated at 37 °C for 1 h. To each tube, 4 ml of 2.5% phosphate-buffered glutaraldehyde was added and mixed gently. After 30 min, samples were centrifuged for 5 min at 500 rpm and washed twice with PBS. One drop of fixed and washed sperm was placed on a silver painted adhesive tape mounted on brass stubs and air dried. 100 Å gold coating was done on Jeol fine coat ion sputter (Jeol, Japan) and the specimens were observed at different magnifications under the scanning electron microscope at sophisticated analytical instrumentation facility, Panjab University, Chandigarh, India.

### Binding of FITC labeled r-SIF to mouse sperm

r-SIF was conjugated with Fluorescein isothiocyanate (FITC) using FITC protein labeling kit (Bangalore Genei, India) according to manufacturer’s instructions. Mouse sperm washed twice with PBS, resuspended in 500 μL of PBS and were incubated with FITC labeled r-SIF. After incubation at 37 °C for 40 min, the sperm were fixed with 3% formaldehyde, washed twice with PBS and observed under fluorescent microscope (Nikon, Japan) at 400X. Sperm incubated with unconjugated FITC dye; unlabeled r-SIF with sperm and sperm suspended in PBS were set as negative control.

### Contraceptive efficacy of r-SIF in mice

Animals were divided into two groups i.e. control and test; six female and three male animals were used for each group. Control mice received 20 μL of PBS whereas different concentration of r-SIF (2.5, 5, 10, 25, 40 and 50 μg; 20 μL of each) was administered in the vagina of test groups. The mice were held in a supine position during the application. All females were immediately mated with proven breeder males in the ratio of 2:1. Mating was confirmed by the presence of a vaginal plug and females were kept under observation for pregnancy outcome. The contraceptive effect of r-SIF was determined based on prevention of pregnancy in test group as compared to controls and the consistency of this response.

## Results

### Construction and screening of genomic library

**Fig. 1:**
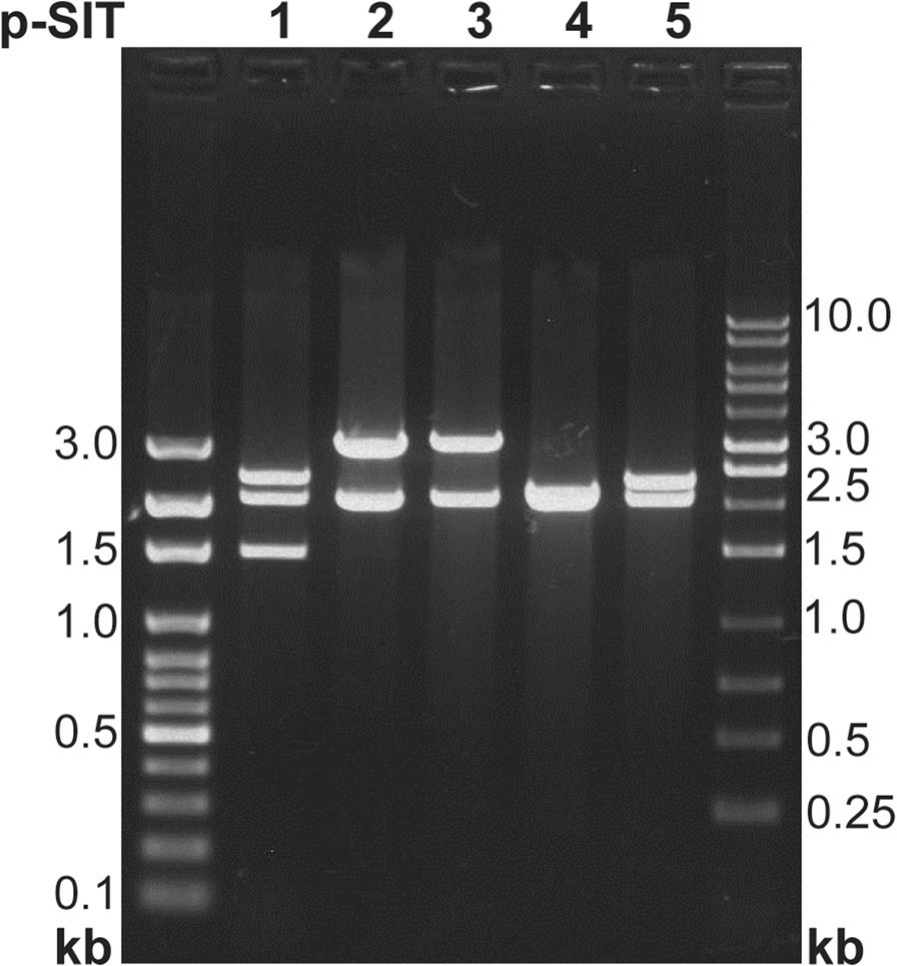
EcoRI-digested pSMART-LCAmp plasmids isolated from transformants carrying *E.coli* DNA fragments (p-SIT) and expressed sperm immobilizing activity. Lanes 1, 2, 3, 4 and 5 correspond to digested plasmid from different transformants, the size of the DNA fragments generated by the digestion correspond to the DNA size ladder.

The genomic library containing 2–6 kb partially digested *E. coli* genomic DNA fragments resulted in five hundred transformants. Screening for SIF positive clones resulted in five positive transformants which exhibited sperm immobilizing activity while *E. coli* DH5α harboring plasmid pSMART did not show sperm immobilizing activity. Recombinant plasmids were recovered from transformed *E. coli* DH5α cells and confirmed for the presence of insert by the digestion (Fig. 1). Plasmid from one transformant designated as sperm immobilizing transformant (SIT-5), showing maximum sperm immobilizing activity in minimum time (5 min) was sequenced.

### Nucleotide sequence analysis of gene encoding the SIF

**Fig. 2:**
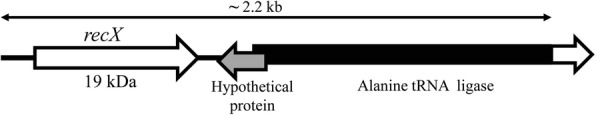
Organization of genes in insert DNA fragment cloned in pSMART-LCAmp.

Recombinant pSMART from transformant SIT-5 subjected to DNA sequencing showed an insert DNA fragment of ~ 2.2 kb. The gene sequence has been submitted to GenBank under accession number MG269998. One ORF of 501 nucleotides was found which showed 100% identity with the regulatory protein RecX of *Enterobacteriaceae* (Fig. 2)*.* The deduced amino acid sequence is 99% identical to RecX protein. The putative RecX consists of 167 amino acids with a theoretical molecular weight of 19.4 kDa. Conclusively, based on NCBI conserved domain database and amino acid sequence similarity, the identified gene belonged to the RecX superfamily [21].

### Expression and purification of recombinant sperm immobilizing factor(r-SIF)

**Fig. 3:**
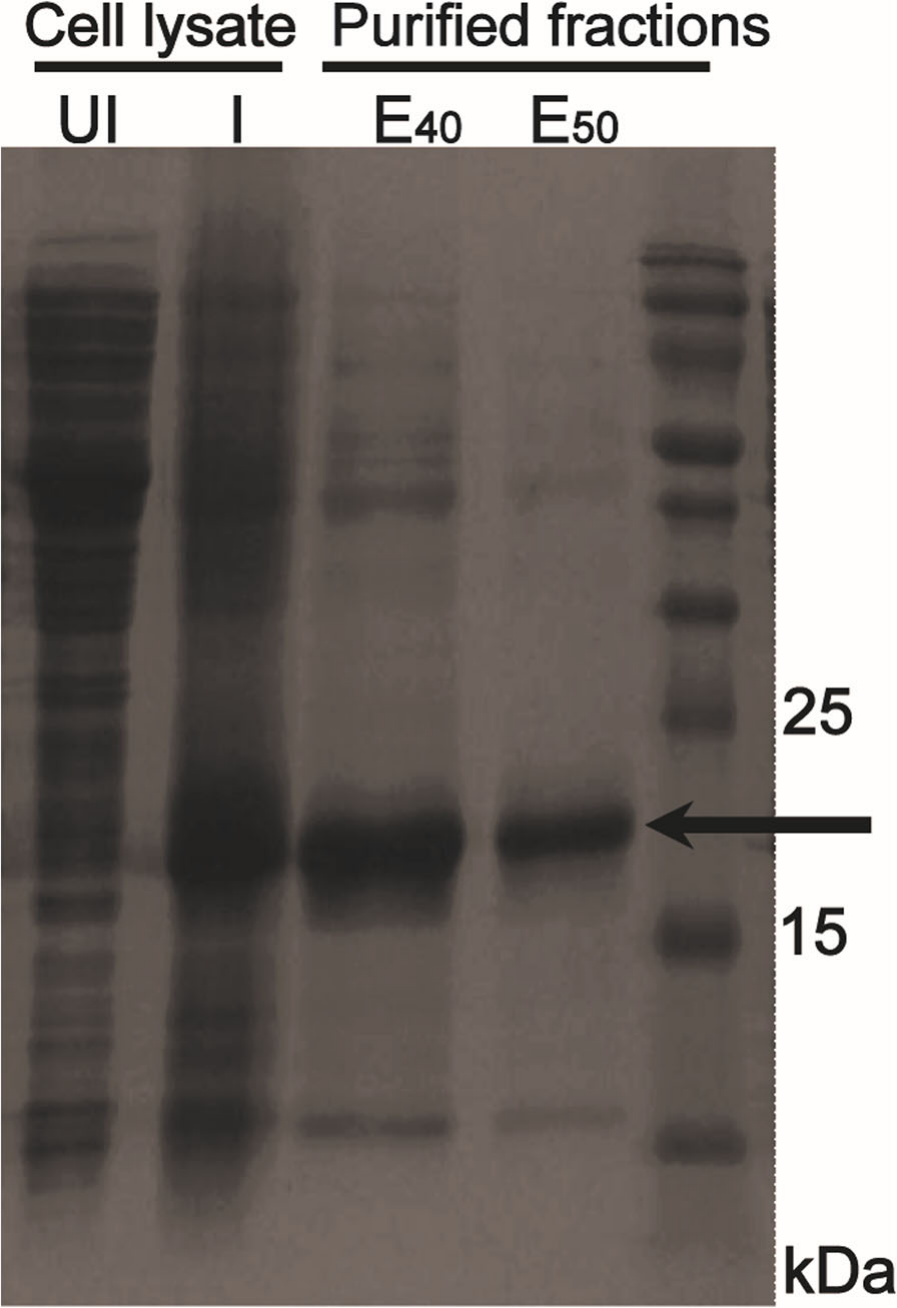
SDS-PAGE analysis of purified RecX: Lane ‘UI’, lysate of un-induced cells from *E. coli* BL21; lane ‘I’, cell lysate of induced cells from *E. coli* BL21; lane ‘E_40_’, fractions containing expressed RecX obtained at 40 mM; lane ‘E_50_’, fractions containing expressed RecX obtained at 50 mM of imidazole.

*E. coli* BL21 cells harboring recombinant plasmid with His-tagged RecX were induced with 0.4% of L-arabinose for protein expression. The recombinant protein was purified by Ni-NTA chromatography and a single protein band between 15 kDa and 25 kDa on comparison to broad range protein marker was obtained in fractions eluted with buffer containing 40–50 mM imidazole (Fig. 3).

### In vitro effect of r-SIF on mouse sperm parameters

**Fig. 4:**
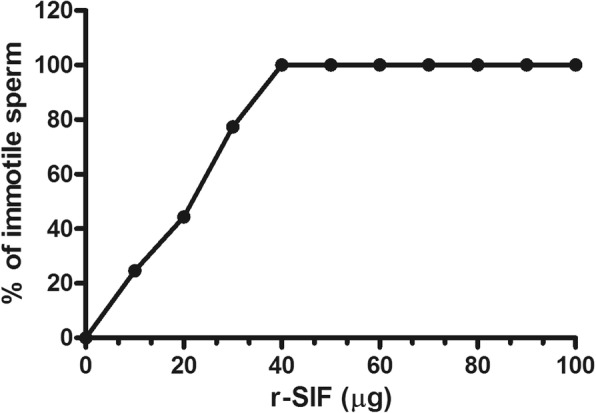
Dose dependent sperm immobilizing activity of recombinant sperm immobilizing factor (r-SIF).

**Fig. 5:**
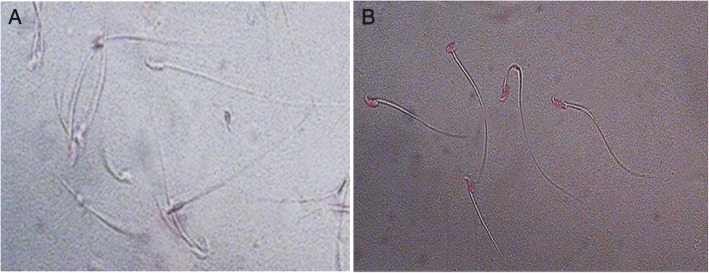
Eosin staining of mouse spermatozoa after 20 s of incubation with (**a**) PBS, unstained head (live) spermatozoa. (**b** Recombinant sperm immobilizing factor, pink stained head (dead) spermatozoa. Magnification for both **a** and **b**: 400X.

**Fig. 6:**
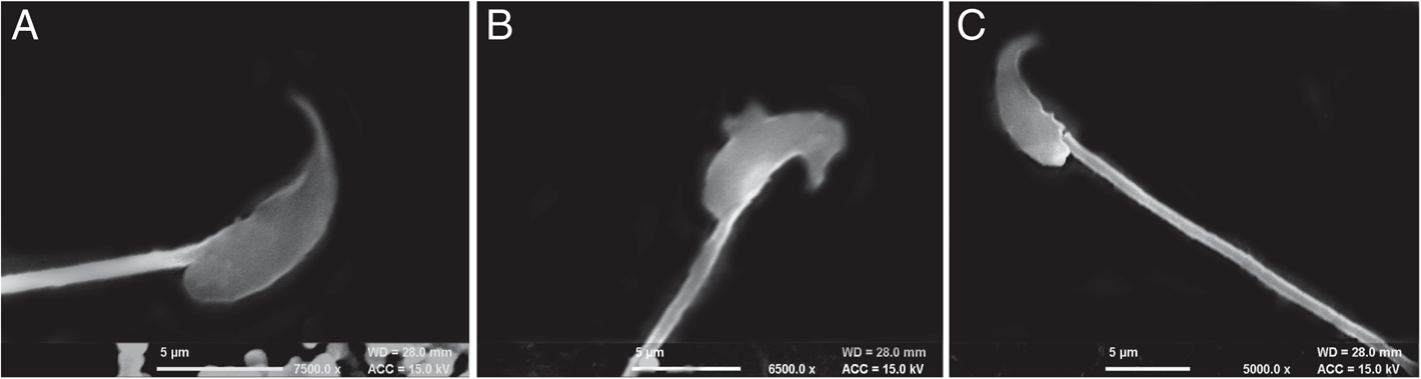
Scanning electron micrograph of mouse sperm upon (**a**) treatment with PBS showed normal morphology at 7500X magnification; (**b**) treatment with recombinant sperm immobilizing factor indicated morphological changes in sperm head membrane at 6500X, and (**c**) detachment of sperm head from mid piece after treatment at 5000X.

- Motility & viability: Complete sperm motility inhibition was observed immediately (within 20 s) with a minimum concentration of 40 μg of r-SIF (Fig. 4). At this concentration, r-SIF not only caused immobilization of spermatozoa but also had a spermicidal effect which was investigated by eosin staining. Pink stained head of dead sperm were distinguished from the unstained head of live spermatozoa (Fig. 5a and b).
- Sperm morphology: The scanning electron micrographs of mouse sperm treated with PBS showed normal morphology, sperm membrane remained smooth and intact all over the whole sperm viz. head, midpiece, and tail (Fig. 6a). In contrast, r-SIF treated mouse sperm resulted in prominent morphological defects, disruption of the head surface membrane (Fig. 6b) and detachment of head region from midpiece (Fig. 6c).

### Binding of FITC labeled r-SIF to mouse sperm

**Fig. 7:**
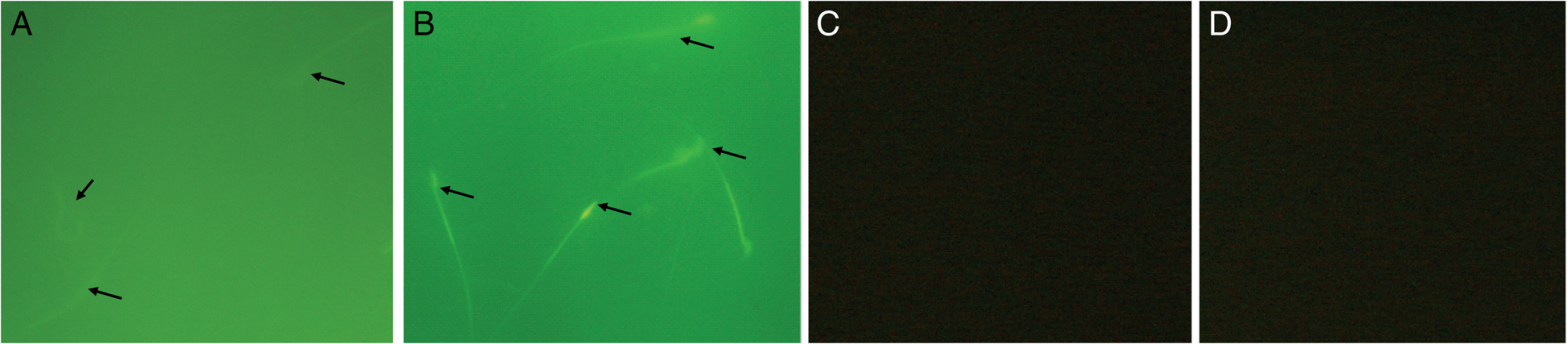
Binding of Fluorescein isothiocyanate labeled recombinant sperm immobilizing factor to mouse sperm. (**a**) Mouse sperm with FITC dye alone showed no florescence from head and neck region of sperm (**b**) Fluorescence was observed on whole sperm. Intensity of fluorescence was more in head and neck regions. Magnification: 400X. (**c**) & (**d**) Mouse sperm incubated with un-labelled r-SIF and sperm suspended in PBS did not show any fluorescence.

Binding of r-SIF to mouse sperm was evaluated by fluorescence microscopy. Incubation of sperm with FITC-r-SIF resulted in staining of the whole sperm viz. head, neck, midpiece, and tail. Head and neck region showed more fluorescence as compared to whole sperm pointing major binding of FITC at these regions (Fig. 7b). In control samples, mouse spermatozoa incubated with FITC dye did not give any fluorescence from head and neck region of mouse spermatozoa as compared to spermatozoa incubated with FITC labeled r-SIF (Fig. 7a). In unlabeled r-SIF mixed with sperm and sperm in PBS there was no fluorescence (Fig. 7c and d).

### Contraceptive effect of r-SIF

**Fig. 8:**
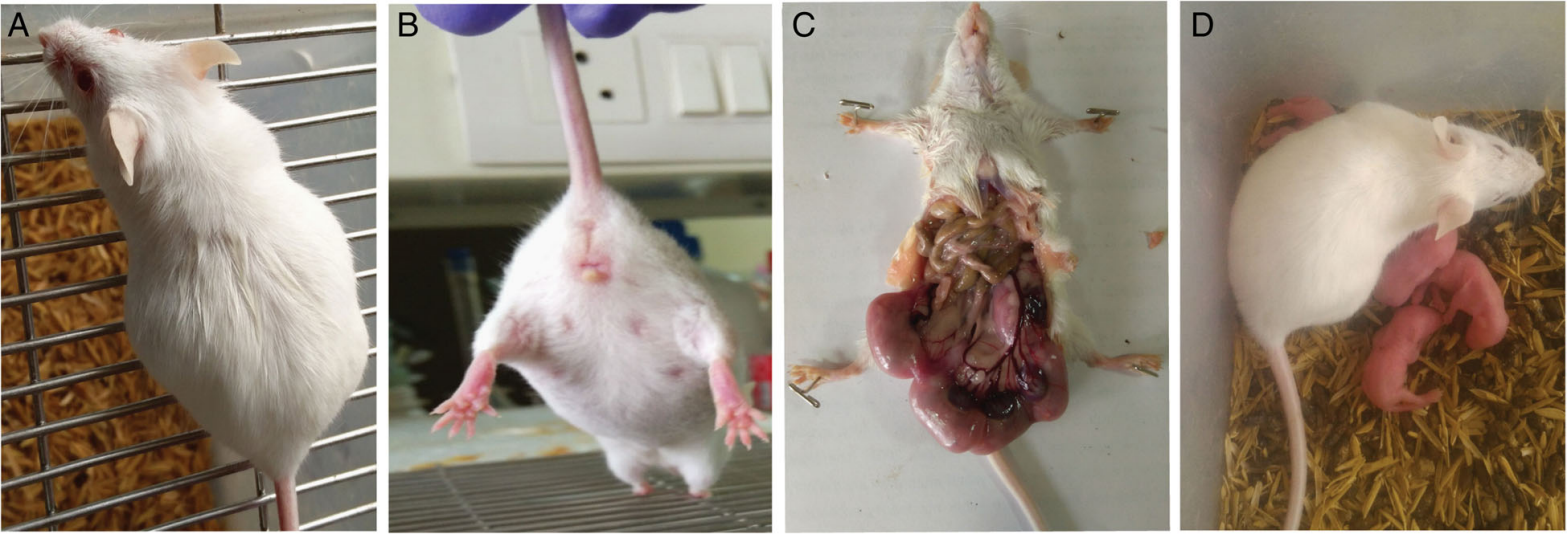
Representative photomicrograph of pregnancy related changes in pregnant mice (**a**) abdominal distension (**b**) protrusion of mammary glands (**c**) string of pearls on day 14 (**d**) pups delivered after 21 days of gestation.

Pregnancy-related changes, i.e., abdominal distension, protrusion of mammary glands, a string of pearls were observed by 12–14 days of gestation period in control group and group received 2.5 μg of r-SIF (Fig. 8a, b, c) whereas these changes were absent in groups treated with ≥5 μg of r-SIF. On the parturition after 21 days, 6–8 pups were delivered by pregnant animals (Fig. 8d), but animals received r-SIF(dose ≥5 μg) did not delivered pups. Hence, a minimum concentration of 5 μg of r-SIF resulted in the complete inhibition of conception. The effect of different doses of r-SIF on fertility outcome is presented in Table 2.

**Table 2:** Effect of r-SIF on fertility outcome in female mice

Dose instilled (20 μl each)	Number of female mice (per group)	Fertility outcome
Placebo (PBS)	6	6/6 (100%)
2.5 μg of r-SIF	6	6/6 (100%)
5 μg of r-SIF	6	0/6 (0%)
10 μg of r-SIF	6	0/6 (0%)
25 μg of r-SIF	6	0/6 (0%)
40 μg of r-SIF	6	0/6 (0%)
50 μg of r-SIF	6	0/6 (0%)

## Discussion

The present-day scenario of increasing population and number of unintended pregnancies looks alarming. Over the years considerable efforts and resources are being spent in controlling the population by available contraceptive methods. Although some progress has been made using these in bringing down the birth rate in urban areas but it has not made much impact on the decrease in overall population. However problems associated with these methods limit their use. Vaginal contraceptives; the topical agents emerge as a replacement to current chemical and physical method of fertility control. However, their mode of action lacks in any cellular specificity and repeated use render the user to discomfort, vaginal irritation and more susceptible to STDs. Several European nations have banned the use of N-9 related compounds due to health risks and potential environmental toxicity [22]. Due to these problems, various products of biological origin which may be either from plant source like CONSAP, NIM-76 or of the microbial source, e.g., Nisin are being developed as vaginal contraceptives.

With an aim to develop safe and effective vaginal contraceptives which not only instantly arrest sperm movement but also overcome the genital epithelial toxicity of available vaginal contraceptives, the present study assessed a recombinant sperm immobilizing factor, produced by *Escherichia coli* for its sperm immobilizing property and contraceptive efficacy in a series of in vitro and in vivo experiments respectively in murine model.

To the best of our knowledge and extensive literature survey, there are no reports about SIF encoding genes. Hence, lack of gene sequence hampered the production of SIF in pure form. In this respect, our approach was to identify SIF encoding gene by the construction of genomic library from *E. coli* genome. Out of approximately five hundred *E. coli* transformants obtained, five transformants expressed the sperm immobilizing activity. SIT-5 showed maximum sperm immobilizing activity indicated by exhibiting its effect on sperm motility in the least time, i.e., 5 min. The nucleotide sequence of the SIT-5 insert showed the presence of a complete ORF, which displayed 100% homology with regulatory protein RecX of *Enterobacteriaceae*, as well as two incomplete ORFs lacking stop codon (Fig.2). The amino acid sequence of the protein product of ORF1 exhibited high sequence identity (99%) with recombination regulator RecX *Escherichia coli*. Further, *recX was* over-expressed in a heterologous host which is a good substitute to overcome the problem of low protein production [23]^.^ *recX* with 6X-his-tag was sub-cloned in pBADmycHisB plasmid downstream of arabinose promoter and transformed in *E. coli* BL21 cells. The optimal expression of tagged RecX was observed with L-arabinose which was purified by Ni-NTA affinity chromatography. The purified factor was homogeneous on SDS-PAGE and its molecular weight was estimated 19 kDa which is close to the expected value of the monomeric form.

In vitro r-SIF showed complete mouse sperm immobilizing activity (within 20 s) at a concentration of 40 μg. In our work, sperm immobilizing activity of *recX* can be related to the antagonistic effect of RecX on RecA activity. *recX* is found just downstream of *recA* gene and act as a repressor of *recA* mediated DNA-dependent ATP hydrolysis, DNA recombinase and co-protease function both in vitro and in vivo in *Escherichia coli* [24]. *recA* reported in *E. coli* has a homolog *rad51* which shares structural similarities and exhibits same comparable molecular functions including DNA dependent ATP-hydrolysis and ATP-dependent recombinase [25–27]. Literature supported that *rad51* has been identified in various higher eukaryotes, including human, mouse, hamster and other higher eukaryotes [28]. For sperm immobilizing activity we hypothesize that as RecX suppresses RecA mediated ATP hydrolysis, most likely it may also inhibit the ATP hydrolysis activity of Rad51. It has been reported that flagellar/sperm movement is driven by dynein motor proteins, which use the energy of ATP hydrolysis to slide the microtubules [29] and absence of ATP hydrolysis inhibits sperm motility. So, the sperm motility inhibition by RecX may be a result of suppression of ATP hydrolysis.

Further experimentation showed that r-SIF exerts spermicidal action confirmed by eosin staining. Pink-headed mouse sperm in test sample confirmed their death as compared to unstained headed live mouse sperm in control. Viability test indicated the rapid sperm damage by r-SIF, which was further validated by scanning electron microscopy. Plasma membrane (PM) plays a vital role in the process of sperm migration and fertilization therefore several spermicidal agents act via structural and functional modulation of the PM, like, disruption of the membrane was observed after exposure of sperm to spermicide [30]. This statement supports the similar obseravtion of our study wherein profound disruption in head membrane and detachment of head were observed during scanning electron microscopy of r-SIF treated sperm. These changes are may be due to loosening of PM, stretching and breakdown of the head membrane. SEM results illustrate that r-SIF exerts spermicidal action by damaging membrane integrity of sperm. Since sperm motility and viability are most essential features for successful fertilization, the spermicidal action of r-SIF makes it a highly desirable candidate for vaginal contraceptive for the future.

Above findings were also suggestive of some interaction between mouse sperm and r-SIF, which were further assessed by fluorescent microscopy. Although fluorescence was observed over the entire surface of the FITC labeled r-SIF sperm, but the intensity of fluorescence was more at head and neck regions indicating the presence of receptors on mouse sperm for r-SIF that might play a role in their interaction. In one of the negative control; sperm and FITC dye, head and neck region of sperm did not show any fluorescence. There was no fluorescence observed with the other two negative controls i.e. sperm incubated with unlabeled r-SIF and sperm suspended in PBS. This indicated that r-SIF specifically binds to mouse spermatozoa at head and neck region.

The contraceptive agents that impair sperm motility in vitro are not necessarily effective in vivo [31]. Therefore, in vivo spermicidal efficacy of r-SIF was assessed using murine model. Fertility outcome experiments showed that animals from groups treated with dose ≥5 μg of r-SIF did not conceive whereas control group animals and group received dose of 2.5 μg delivered the pups. Our studies suggest, r-SIF effectively blocks sperm motility and prevents the establishment of pregnancy in mice. These results are in agreement with studies done by Reddy *et al*. where they reported contraceptive potential of a microbial compound Nisin [13] and with earlier studies of our laboratory which demonstrated the contraceptive potential of a microbial factor [32].

## Conclusion

Our study concludes that RecX is an efficient spermicidal agent which effectively prevents pregnancy in mice and could be developed as a potential vaginal contraceptive for future use in women. Further studies in this direction are under way to evaluate the safety of r-SIF in mouse model.

## Abbreviations

FITC: Fluorescein isothiocyanate
MEC: Minimum effective concentration
r-SIF: Recombinant sperm immobilizing factor
SEM: Scanning electron microscopy
SIF: Sperm immobilizing factor
SIT: Sperm immobilizing transformant
